# New Developments at "The London": Mr. W. T. Paulin on the Effect of the Insurance Act

**Published:** 1914-06-13

**Authors:** 


### NEW DEVELOPMENTS AT "THE LONDON."

### Mr. W. T. Paulin on the Effect of the Insurance Act.

Mr. W. T. Paulin, who took the chair at the quarterly court and is the treasurer of the London Hospital, compared the work of this last quarter with the same quarter of last year. On one day, he said, in this quarter there were 864 patients in the beds—a record. The highest number during the second quarter of last year was 848.

This year there have been 823 beds on an average in daily occupation, as against 790 to the same date in 1913. To have 33 more beds in daily occupation is serious, because from the financial point of view an occupied bed costs £2 3s. 6d. a week.

There are no evidences that there has been more than the usual sickness about. The out-patients, he added, may help to explain the increase. The casualty out-patients have gone down. They have fallen to 18,400 in the last three months, from 19,700 for the same three months last year.

This was what one would expect as the Insurance Act got into regular work. Trivial casualty cases are referred to the doctors on the panel. The hospital did not profit much, however, by such an arrangement. These casualty patients were not *costly* patients, and no member of our staff could be cut off because of this slight reduction. But while casualties have, as he expected, decreased, ordinary out-patients have increased from 17,000 to 20,000 during the same two periods. There was an increase also in the seriousness of the ailments. In every 100 out-patients they used to admit to the beds before the Act came into force—7; now 11 out of every 100 out-patients were admitted. The number of cases sent up by doctors for further assistance and treatment has also increased from 7 per cent. to 13 per cent.

The effect of the Act seems to be, therefore, said Mr. Paulin, that cases are found to be suffering from some ailment which before the Act was neglected and overlooked, and the patients come to hospital for treatment earlier. Though this is indeed good for the patient, it enormously increases the work of the hospital officials and the expenditure. Of out-patients who are over fourteen years of age 32 per cent. (roughly, 1 in 3) is an insured patient, and the hospital is treating him on behalf of the State. And yet, with two exceptions, no Friendly Society has made any contribution to the hospital for this immense amount of work done on behalf of its members.

Another way of showing the increased pressure, he added, was this: Every bed in the hospital now has 21 patients passing through it throughout the year. The previous year it was 19; before that 18; and, until a few years ago, it was 12. At that time the average stay of a patient in hospital was a month; it is now 17 days.

[The report itself appeared last week.]

